# Exploring the Effect of Parental Styles on Social Skills: The Mediating Role of Affects

**DOI:** 10.3390/ijerph19063295

**Published:** 2022-03-10

**Authors:** Carlos Salavera, Pablo Usán, Alberto Quilez-Robres

**Affiliations:** Department of Psychology and Sociology, Faculty of Education, University of Zaragoza, 50009 Zaragoza, Spain

**Keywords:** parenting styles, affects, social skills

## Abstract

Parental educational styles have a significant effect in personal development. These styles (authoritative, democratic, permissive and neglectful) can be related to affects and social skills at the individual level. The study presented here, which comprised 456 participants (151 men; 33.11%), with an average age of 22.01 years (*s.d.* = 2.80), aimed to analyse the relationship between parental styles, affects and social skills, as well as the role played by affects in the relationship between parental style and social skills. The results suggest that the constructs under study are closely related. The most common parental style is democratic. By gender, permissive styles were more often applied to women and authoritative styles to men. No significant gender differences were found in the application of democratic and neglectful parental styles. In terms of emotional support, women were found to have higher negative affect scores and men higher emotional support scores. People with parents that use democratic and permissive styles scored higher in all variables related to affects and social skills, which challenges the notion that democratic styles are the best parental styles in terms of socialisation of children. The results of the affect and social skills scales were analysed in relation to parenting styles, indicating that children educated under a democratic parental regime tend to yield higher scores in terms of social skills than children educated under any other form of parental regime and medium scores in terms of affects. Finally, it was found that parenting styles have a direct influence on social skills, which tend to improve when affects play a mediating role between these two constructs. These results suggest that parenting styles are closely related to affects and social skills. In addition, they also suggest that affects play a mediating role in the relationship between parenting styles and social skills. Finally, owing to the impact that parenting styles have on affects and social skills, more research is needed to address this issue.

## 1. Introduction

Family plays a crucial role in the early acquisition of habits, skills and behaviours. Adults, in both families and the school, are essential in the education of children [1,2,3,4]. Individual and contextual factors also play a direct role in educational processes.

Complementing Baumrind’s [5,6] pioneering research on parental styles and the effect of family socialisation on social skills in children and teenagers, MacCoby and Martin [7] developed a typology of four parental styles: authoritative, democratic, permissive and neglectful. These styles result from the combination of two variables: affects and control. As such, parenting styles can be defined as the behaviour of adults as children’s models in terms of everyday choices, decision making, conflict resolution, expectation management and rulemaking. These will determine the children’s behaviours and emotions throughout their lives [8,9].

The socialisation strategies mobilised by parents for the social development and integration of their children can be characterised based on the following criteria: (1) communication levels (acceptance–rejection, warmth–coolness, proximity–distance); (2) the tone of the relationship (affection–hostility); (3) the tools used to channel behaviour (autonomy–control, flexibility–rigidness, permissiveness–restrictions). The combination of these variables results in different parenting styles which, in any case, are only general behavioural trends, because the parent–children relationship is bidirectional [10,11]. As such, although there is some consensus about the division of parenting styles into four broad styles (democratic, authoritative, permissive and neglectful), the norm is for these to mix and evolve according to developments in the family relationship.

The democratic style is characterised by open shows of parental affection, giving explanations, expressing concern for the needs of the children, promoting desirable behaviours, justifying reprimands and communicating openly. These households are dominated by a democratic environment and emotional warmth. As a result, the children tend to develop good social skills, self-control, initiative, motivation, self-esteem, good morale and realistic self-concept and are generally happy, spontaneous, reliable, committed (altruism, solidarity), sociable, both within and outside the household, prone to achieve and unlikely to cause parent–children conflicts [2,3,4,12].

The authoritative style is characterised by detailed and rigid rules, prioritising punishment over praise, blaming children for mistakes, closed and unidirectional communication (no dialogue), frequent asserting of parental authority and an autocratic environment. As a result, children have little autonomy and self-confidence, poor social skills, low creativity, they are prone to aggression and impulsiveness and tend to adopt heteronomous moral standards (avoidance of punishment) and they are less happy and spontaneous [2,3,4,13,14,15].

The permissive style is characterised by a lack of concern for the children’s negative or positive behaviour, passiveness, the children’s misbehaviour tends to go unpunished, all the children’s impulses are tolerated and authority is insufficiently asserted, no restrictions are imposed and the wishes of children are easily granted. As a result, children tend to develop poor social skills, low self-esteem weak self-identity poor self-control and hetero-control, lack of emotional stability, negative self-concept, poor self-concept and self-responsibility, insecurity, little regard for the rules and for others and academic underachievement [2,3,4,16,17].

The neglectful style is characterised by emotional indifference towards the children’s issues, parental relinquishment of responsibility, lack of motivation, commitment and involvement and immaturity. As a result, children develop poor social skills, impulsivity and aggression and tend to lack motivation, commitment and maturity [2,3,4,18,19].

To some extent, these parenting styles, along with other household factors, such as the school environment, relation with peers and individual traits such as personality and social skills, determine the individual’s behaviour [20,21,22,23,24,25,26].

On the other hand, affects are defined as a binary relationship between positive and negative emotions, which is grounded, according to Watson and Tellegen [27], on a hereditary base. According to this view, positive affects relate to pleasant emotions: motivation, affiliation, achievement and success. Negative affects, for their part, relate to unpleasant emotions: fear, inhibition, insecurity, frustration and failure [28,29,30]. In this way, a person dominated by positive affects generally harbours positive feelings such as satisfaction, enthusiasm, energy, friendship, attachment, affirmation and trust. They are, therefore, extroverted, optimistic and resilient. Conversely, a person dominated by negative affects tend to harbour negative feelings such as detachment, boredom, sadness, guilt, shame and envy. They are, therefore, prone to react to negative stimuli aggressively and to emotional lability, stress and negative views [31,32,33].

Finally, social skills can be defined as a set of abilities used in interpersonal relationships and interactions. They determine a person’s ability to act in a way that leads to rewards and avoids punishment and social ostracism [34,35,36]. That is, they are a set of skills that expresses an individual’s feelings, attitudes, wishes and opinions in an interpersonal setting. Good social skills tend to solve immediate interpersonal conflicts and minimise the chance for future confrontations [37,38]. These skills are chiefly acquired through training, observation, imitation, trial and information; that is, they are acquired traits. Nobody is born with a given repertoire of social skills; they are learned behaviours. There are two major types of social skills, basic and complex, and the former needs to be learned before the latter can be acquired. The learning process begins during childhood and develops largely during adolescence, when adult communicational and relational skills are acquired. Social skills are a necessary tool for positive social relations to lead to personal wellbeing [39,40].

### Parenting Styles, Affects and Social Skills

Parenting styles, affects and social skills play a crucial role in personal development and in the way individuals handle themselves in social contexts.

Affects and social skills can act as risk or protection factors with regard to problematic behaviours during adolescence. Increasing personal autonomy, changes in family relationships, the transition from specific to formal thinking, shifting social relations, etc., are factors that shape the psychosocial development of the individual, in which the family plays a central role. Parenting styles and family relationships around adolescents are a key factor in their emotional, social and personal development [4,41]. These notions (parenting styles, affects and social skills) can be related, with the parenting styles having an effect on the other variables.

Finally, it is worth mentioning that parenting styles will have different effects on each individual, based on their personal traits. That is, there is no correct parenting style, which must instead adapt to the individual traits and environmental conditions, as pointed out by Aroca and Cánovas [42].

The first aim of this study is to analyse the relationship between parenting styles, affects and social skills. The research also aims to assess the role of gender in parenting styles, social skills and affects. Several lines of reasoning led us to this expectation. First of all, some authors have pointed out how the parenting styles used with men and women are different [43,44]. Second, although some studies reported that women report higher levels of social skills and are more concerned about the quality of their interpersonal relationships, others point to the opposite [45,46]. Finally, women tend to score higher than men on tests of positive and negative affects [47,48]. Although empirical studies have found these gender differences separately in each of the variables studied, there is no empirical evidence of how these gender differences can influence the relationship of parenting styles with social skills and affects. So, for the investigation, we took an exploratory approach to gender differences in the association between these three variables.

The two starting hypotheses are as follows: (1) parenting styles, affects and social skills are related; (2) affects will perform as a mediating variable in the relationship between parenting styles and social skills.

## 2. Materials and Methods

### 2.1. Participants

The sample comprised 456 participants from the city of Zaragoza (teaching degree students, University of Zaragoza). All participants were volunteers. The average age of participants was 22.01 years (*s.d.* = 2.80). All participants signed an informed consent form, and all the ethical considerations set out in the Declaration of Helsinki were met. The investigation was conducted according to the guidelines of the Declaration of Helsinki and approved by the Ethics Review Committee of the OPIICS research group (S46_20R), Psychology and Sociology Department, Universidad de Zaragoza, on 26 February 2021. The study met all ethical criteria for research with human beings (informed consent, right to information, full confidentiality, non-discrimination, free participation and the right to abandon the study at any point). Representativeness tests were undertaken; the confidence level was 99% and the sampling error 5%. It was concluded that the sample was representative of the province of Zaragoza. The study adopted an ex post facto research design [49].

### 2.2. Instruments

#### 2.2.1. Parenting Styles

Data concerning parenting styles were collected using a multifactor children adaptation self-assessment test (TAMAI) [50]. TAMAI is a 175-item self-evaluation test used to assess maladaptation. The first-tier scales are as follows: (1) personal maladaptation; (2) school maladaptation; (3) social maladaptation; (4) family dissatisfaction; (5) father–mother style; (6) parenting style discrepancies; (7) reliability criteria or contestation style (pro-image criteria and contradiction criteria). In this study, only the 78 items that address parenting styles were used, specifically the father–mother scale, which assesses the children’s view on parenting styles. Following previous studies [51], participants were asked to respond retrospectively to parenting-style-related items with reference to their own childhood. The study yielded a Cronbach’s alpha coefficient of 0.87 (Ω = 0.88).

#### 2.2.2. Positive and Negative Affects PANAS Scale

The PANAS [30] is a 20-item Likert scale, in which 10 items refer to positive affects (AP) and 10 to negative affects (AN); the responses range from 0 (absence of emotion) to 5 (strong presence of emotion). The scale yielded a Cronbach’s alpha coefficient of 0.88 for positive affects (Ω = 0.88) and of 0.86 for negative affects (Ω = 0.87).

#### 2.2.3. ICQ-15 Social Skills Questionnaire

This questionnaire ICQ-15 [52] assesses the multi-factor social skills construct, based on five different but interrelated scales. They assess the individual’s skill in terms of: (1) beginning relationships; (2) negative assertion; (3) revealing personal information; (4) providing emotional support; (5) handling interpersonal conflicts. It is a 15-item, 5-point Likert scale. The Spanish version validated by Salavera and Usán [53] was used. For this study, the scale yielded a Cronbach’s alpha coefficient of 0.84 (Ω = 0.85).

### 2.3. Protocol

The principal investigator explained the main aim of the study to the participants, emphasising the importance of answering all items when the questionnaires were handed out.

Participants were given 45 min to complete the questionnaires and the informed consent form. Participants were reminded that responses were to be kept anonymous and confidential. The data were collected in April and May 2021.

The data were processed with SPSS 26.0 statistical software. After performing normal distribution and equality of variances tests, we decided to use parametric techniques. Basic descriptive analysis of mean trends (average), percentages, frequencies and dispersion (standard deviation) was undertaken for each variable. Student’s *t*-test was also used on independent samples as a way to establish average differences with continuous and normal variables. In all cases, we used the lowest significance level possible, and all differences *p* < 0.05 were regarded as significant. Cohen’s *d* was used to analyse the size of the effect and thus the magnitude of the differences found with Student’s *t*. Following Cohen [54], effect size can be regarded as follows: *d* = 0.20 (small), *d* = 0.50 (moderate) and *d* = 0.80 (large). Interactions were created following Aiken and West [55] and Campbell and Kashy [56]. In order to aid the interpretation, an effect (–1, 1) code was used for binary variables. Average conglomerates were estimated in order to create clusters of participants based on parenting styles and the affect and social skills scores. Finally, mediation analyses were undertaken to establish if affects play a mediating role in the relationship between parenting styles and social skills, following Baron and Kenny [57].

## 3. Results

The distribution of the participants in the four parenting styles is detailed in Table 1. The democratic and permissive styles turned out to be the most used in the sample. By gender, the democratic and negligent styles were used in the same proportion in men and women. However, the permissive style was proportionally more used in women (28.6% in women vs. 18.5% in men), while the opposite situation was found in the authoritarian style (27.8% in men vs. 20.1% in women).

Next, the scores obtained by the participants on the scales that measure affect and social skills were analysed (Table 2). Differences were only found in negative affects (*p* = 0.002; *d* = −0.311), with higher scores in the case of women. In social skills, men obtained higher scores in the factor corresponding to self-reveal (*p* = 0.011; *d* = 0.141).

Following this, the results of the affect and social skills scales were analysed in relation to parenting styles. The results (Table 3) reveal significant differences. Children educated under democratic and permissive regimes scored higher in all affect- and social-skills-related variables. Children educated under a democratic regime scored higher in terms of positive affects and those variables in the emotional skill scale related to self-reveal, emotional support, negative assertion and initiation. Children educated under a permissive regime scored higher in terms of negative affects and conflict management.

Afterwards, the scores were divided into three groups, based on averages and standard deviations (low, medium and high). Table 4 shows that democratic parenting styles lead to high positive affects and social skills, permissive parenting styles result in high negative affects (up to one in five participants educated under a permissive regime scored high in this variable), emotional support and social skills, authoritative parenting styles lead to very high scores in either positive or negative affects and finally, neglectful parenting styles result in low average scores in the variable positive affects and all the variables related to social skills.

Finally, in order to test the second hypothesis, mediation analysis was carried out following Baron and Kenny [57]. After ensuring that the study met all the requisites pointed out by these authors, Hayes’s [58] SPSS 24.0 d Process 3.5 macro was used. The mediation analysis took into consideration gender and age variables, which were found not to have a significant effect on the relationship between parenting styles and social skills.

**Figure 1:**
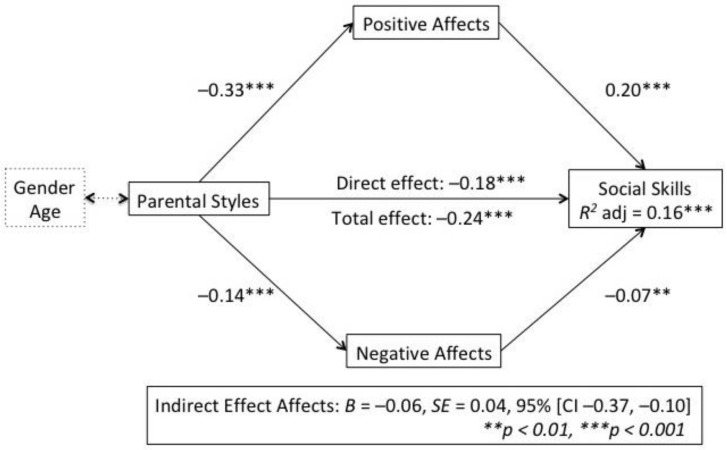
Simple mediation model of positive and negative affects on the relationship between parenting styles and social skills, accounting for age and gender.

In order to determine that the mediation effect was statistically significant, bootstrapping analysis (10,000 runs) and Sobel’s test were conducted. It was found that positive, but not negative, affects play a mediating role in the relationship between parenting styles and social skills. The results suggest that parenting styles (VI) are a mediating variable (VM) on positive affects (−0.33) and on negative affects (−0.14) (in both cases *p* < 0.001). In addition, social skills are a mediating variable (VD) on positive affects (0.20) and negative affects (−0.07). Zero was not included in the bootstrap interval, IC 95% [−0.37, −0.10], and the result of Sobel’s test indicates that the value of c’ is statistically significant (*z* = 2.41; *p* = 0.005); therefore, the mediation effect can be said to be total (Figure 1).

Following the hypothesis, it was found that educational styles have a positive effect on social skills (−0.18) (*p* < 0.001) and a total effect (direct + indirect effect) of −0.06 (*p* < 0.001), mediated by positive affects, which demonstrates that affects play a mediating role in the relationship between parenting styles and social skills. The proportion of variance for social skills as explained by the model was *R^2^* = 0.16.

## 4. Discussion

The aim of this study was to analyse the relationship between parenting styles, affects and social skills. The study was based on two starting hypotheses: (1) parenting styles, affects and social skills are related; (2) affects play a mediating role in the relationship between parenting styles and social skills.

Gender differences are among the most widely studied factors in these relationships [43,44]. Some studies suggest that fathers are more prone to authoritative parental styles and mothers to more inductive styles [59,60,61,62,63,64]. Our results indicate that democratic parenting styles are adopted by a similar percentage of fathers and mothers. Meanwhile, permissive styles are more often applied to women and authoritative styles to men. Finally, neglectful parenting styles are somewhat more commonly applied to men. This could be the result of a greater percentage of men presenting behavioural problems, forcing parents to adopt disciplinary measures [65] and apply punishments [62,66]. Our results indicate that affects and social skills are related to parenting styles. Previous studies have pointed out that parental affection is related to children’s psychological wellbeing [18,67,68,69], and this was confirmed by our results. We also attested higher scores in terms of negative affects among women, as also pointed out in previous studies [70] that yielded higher scores in the social-skills-related variable emotional support, emphasising the important role played by affect in social skills, as noted in the existing literature [71,72,73].

The results were divided into three groups based on averages and standard deviations (low, medium and high). Table 4 shows that democratic parenting styles are related to medium scores but higher than those yielded by other parenting styles in terms of positive affects, and this agrees with the idea that consistent rulemaking leads children to not perceive parental authority as rigid and to comply with rules voluntarily, resulting in high scores in terms of self-concept, self-esteem and social skills [7,74,75,76], even if the notion that democratic parenting styles offer the best tools for the socialisation of children has been challenged [77,78,79]. Permissive parenting styles, on the other hand, result in higher scores in terms of negative affects than the other parenting styles, and one out of five of the respondents educated under a permissive regime scored high in this variable. In addition, people educated under a permissive regime scored high in terms of emotional support, as suggested by some studies that argue that permissive parenting styles offer better chances of psychosocial fit to children than democratic styles [80,81,82]. Authoritative parenting styles lead to higher scores in either positive or negative affects than those yielded by democratic and permissive parenting styles. This agrees with the idea that authoritative parenting styles are related to such aspects as low self-esteem and self-concept and poor social skills [83,84]. Finally, neglectful parenting styles lead to low scores in positive affects and all variables related to social skills. These results suggest that neglectful parenting styles have a negative effect in the socialisation of children, wellbeing, self-esteem, autonomy and social skills [18,75], as our results confirm.

In addition, in order to test the second hypothesis, the possible mediating effect of affects on the relationship between parenting styles and social skills was examined. As previously noted, gender and age variables were taken into account and were found not to have a significant impact on the relationship between parenting styles and social skills. The results indicate that parenting styles have a mediating effect on positive and negative affects, strongly suggesting that parenting styles play a significant emotional role, in line with previous studies, which have related parenting styles to other variables such as subjective wellbeing [85,86]. In addition, the study shows that greater scores in terms of positive affects go together with better social skills and vice versa. Therefore, the study confirmed the second hypothesis; the results indicate that the mediating effect is total, demonstrating the mediating role played by affects in the relationship between parenting styles and social skills. Although this relationship was already suggested by previous studies [87,88], none of these studies undertook a holistic approach such as the one adopted here.

The limitations of the study are as follows. First, it has a lateral design, which precludes causal relationships from being established. In addition, as the sample involved a very specific social group (local teaching degree university students), it is risky to extrapolate the results. Finally, the data were based on self-assessment questionnaires and, such as with all these sorts of study, social conformity issues could introduce bias to the results. Additional studies should relate the results with those yielded by broader and more detailed questionnaires, ideally including qualitative evidence. Family models should also be taken into consideration, namely: (1) nuclear families; (2) extended families; (3) simultaneous or superimposed families; (4) single-parent and/or large families; (5) childless couples; (6) communal families; (7) other forms of family organisation; (8) foster homes; (9) domestic units [89]. Therefore, future research should explore the interaction of, and the complex relationships between, these variables (type of family, parenting styles, affects and social skills). However, our study leads to clear conclusions concerning the constructs under consideration and emphasises the need to implement specific educational programmes to improve them, especially concerning parenting styles, which have major implications in terms of personality development.

## 5. Conclusions

The study suggests that parenting styles are related to affects and social skills. The study also indicates that affects play a mediating role in the relationship between parenting styles and social skills. Finally, owing to the implications of parenting styles not only for affects and social skills but for the overall psychological, social and personal development of children, it is concluded that these issues should be addressed jointly by families and schools. Our results encourage us to keep searching for new questions, methodologies and answers with which to contribute to the positive socio-emotional development of the person.

## Figures and Tables

**Table 1:** Parenting styles by gender.

Parenting Styles	Men	Women	Total
Democratic	57 (37.7%)	118 (38.8%)	175 (38.5%)
Permissive	28 (18.5%)	87 (28.6%)	115 (25.3%)
Authoritative	42 (27.8%)	61 (20.1%)	103 (22.6%)
Neglectful	24 (15.9%)	38 (12.5%)	62 (13.6%)
Total	151 (100%)	304 (100%)	455

**Table 2:** Affect and social skills questionnaire results.

Variables Research	Men		Women		*t*	*p*	*d* Cohen
	*x*	*ds*	*x*	*ds*			
Positive affects	18.34	2.77	18.34	2.85	0.001	0.978	0.010
Negative affects	12.08	3.23	13.05	3.00	10.12	0.002	−0.311
Initiation	10.16	2.68	9.93	2.87	0.669	0.504	0.082
Negative assertion	7.00	1.93	6.9	1.96	0.406	0.100	0.051
Self-reveal	10.96	2.44	10.7	2.45	2.55	0.011	0.032
Emotional support	12.77	1.96	12.46	2.40	1.12	0.260	0.141
Conflict management	10.49	2.38	10.67	2.31	−0.614	0.540	−0.076

**Table 3:** Affect and social skills scores by parenting style.

Variables Research	Democratic	Permissive	Authoritative	Neglectful	Average
Positive affects	**18.69**	18.47	17.97	17.72	18.34
Negative affects	12.80	**13.00**	12.55	12.35	12.73
Self-reveal	**11.03**	10.82	10.40	9.72	10.66
Emotional support	**12.96**	12.94	12.73	12.41	12.83
Negative assertion	**10.97**	10.82	10.62	10.20	10.75
Initiation	**10.37**	10.21	10.26	9.61	10.20
Conflict management	10.88	**10.96**	10.49	10.25	10.73

Note: Values in bold represent the highest score for each category.

**Table 4:** Percentage of participants by group and parenting styles.

Variables Research	Level	Democratic	Permissive	Authoritative	Neglectful	Total
Positive affects	Low	21 (12.0%)	15 (13.0%)	20 (19.4%)	**16 (25.8%)**	72 (15.8%)
	Medium	**129 (73.7%)**	84 (73.0%)	68 (66.0%)	38 (61.3%)	319 (70.1%)
	High	25 (14.3%)	16 (13.9%)	**15 (14.6%)**	8 (12.9%)	64 (14.1%)
Negative affects	Low	23 (13.1%)	15 (13.0%)	**18 (17.5%)**	9 (14.5%)	65 (14.3%)
	Medium	122 (69.7%)	77 (67.0%)	66 (64.1%)	**46 (74.2%)**	311 (68.4%)
	High	30 (17.1%)	**23 (20.0%)**	19 (18.4%)	7 (11.3%)	79 (17.4%)
Self-reveal	Low	22 (12.6%)	17 (14.8%)	16 (15.5%)	**21 (33.9%)**	76 (16.7%)
	Medium	127 (72.6%)	84 (73.0%)	**81 (78.6%)**	37 (59.7%)	329 (72.3%)
	High	**26 (14.9%)**	14 (12.2%)	6 (5.8%)	4 (6.5%)	50 (11.0%)
Emotional support	Low	16 (9.1%)	11 (9.6%)	15 (14.6%)	**10 (16.1%)**	52 (11.4%)
	Medium	**113 (64.6%)**	69 (60.0%)	63 (61.2%)	38 (61.3%)	283 (62.2%)
	High	46 (26.3%)	**35 (30.4%)**	25 (24.3%)	14 (22.6%)	120 (26.4%)
Negative assertion	Low	30 (17.1%)	21 (18.3%)	19 (18.4%)	**18 (29.0%)**	88 (19.3%)
	Medium	115 (65.7%)	**79 (68.7%)**	70 (68.0%)	36 (58.1%)	300 (65.9%)
	High	**30 (17.1%)**	15 (13.0%)	14 (13.6%)	8 (12.9%)	67 (14.7%)
Initiation	Low	25 (14.3%)	13 (11.3%)	16 (15.5%)	**14 (22.6%)**	68 (14.9%)
	Medium	109 (62.3%)	**86 (74.8%)**	65 (63.1%)	42 (67.7%)	302 (66.4%)
	High	**41 (23.4%)**	16 (13.9%)	22 (21.4%)	6 (9.7%)	85 (18.7%)
Conflict management	Low	22 (12.6%)	13 (11.3%)	16 (15.5%)	**18 (29.0%)**	69 (15.2%)
	Medium	115 (65.7%)	78 (67.8%)	**71 (68.9%)**	34 (54.8%)	298 (65.5%)
	High	**38 (21.7%)**	24 (20.9%)	16 (15.5%)	10 (16.1%)	88 (19.3%)

Note: Values in bold are the highest percentages in each category.

## Data Availability

The data presented in this study are available on request from the corresponding author.

## References

[1] Cámara A., López J.B. (2011). Estilos de educación en el ámbito familiar. Rev. Española Orientac. Psicopedag..

[2] Doinita N.E., Maria N.D. (2015). Attachment and parenting styles. Procedia-Soc. Behav. Sci..

[3] Fan J., Chen B.B. (2020). Parenting styles and co-parenting in China: The role of parents and children’s sibling status. Curr. Psychol..

[4] Newman B.M., Newman P.R. (2020). Theories of Adolescent Development.

[5] Baumrind D. (1967). Child care practices anteceding three patterns of preschool behavior. Genet. Psychol..

[6] Baumrind D. (1971). Current patterns of parental authority. Dev. Psychol..

[7] Maccoby E.E., Martin J.A., Mussen P.H., Hetheringtono E.M. (1983). Socialization in the context of the family: Parent-child interaction. Handbook of Child Psychology: Vol. IV—Socialization, Personality and Social Development.

[8] Jorge E., González M.C. (2017). Estilos de crianza parental: Una revisión teórica. Inf. Psicol..

[9] Morris A.S., Ratliff E.L., Cosgrove K.T., Steinberg L. (2021). We know even more things: A decade review of parenting research. J. Res. Adolesc..

[10] Bocanegra E. (2007). Las prácticas de crianza entre la Colonia y la Indepen-dencia de Colombia: Los discursos que las enuncian y las hacen visibles. Rev. Latinoam. Cienc. Soc. Niñez Juv..

[11] Burke J.D., Pardini D.A., Loeber R. (2008). Reciprocal relationships between parenting behavior and disruptive psychopathology from childhood through adolescence. J. Abnorm. Child Psychol..

[12] Miklikowka M., Hurme H. (2011). Democracy begins at home: Democratic parenting and adolescents’ support for democratic values. Eur. J. Dev. Psychol..

[13] Bi X., Yang Y., Li H., Wang M., Zhang W., Deater-Deckard K. (2018). Parenting styles and parent–adolescent relationships: The mediating roles of behavioral autonomy and parental authority. Front. Psychol..

[14] Li G., Wang B. (2016). Effect of authoritative parenting and family relation on adolescent leadership: The mediating role of general self-concept. Int. J. Psychol..

[15] Lavric M., Naterer A. (2020). The power of authoritative parenting: A cross-national study of effects of exposure to different parenting styles on life satisfaction. Child. Youth Serv. Rev..

[16] Calders F., Bijttebier P., Bosmans G., Ceulemans E., Colpin H., Goossens L., Van Den Noortgate W., Verschueren K., Van Leeuwen K. (2020). Investigating the interplay between parenting dimensions and styles, and the association with adolescent outcomes. Eur. Child Adolesc. Psychiatry.

[17] Kuppens S., Ceulemans E. (2019). Parenting styles: A closer look at a well-known concept. J. Child Fam. Stud..

[18] Torío S., Peña J.V., Rodríguez M.C. (2008). Estilos educativos parentales: Revisión bibliográfica y reformulación teórica. Teoría Educ..

[19] Wong T.K.Y., Konishi C., Kong X. (2021). Parenting and prosocial behaviors: A meta-analysis. Soc. Dev..

[20] Bagán G., Tur-Porcar A.M., Llorca A. (2019). Learning and Parenting in Spanish Environments: Prosocial Behavior, Aggression, and Self-Concept. Sustainability.

[21] Fan W.Q., Li M.T., Chen X.Y. (2021). Reciprocal relationship between parenting styles and interpersonal personality in Chinese adolescents. Front. Psychol..

[22] Gallarin M., Alonso-Arbiol I. (2012). Parenting practices, parental attachment and aggressiveness in adolescence: A predictive model. J. Adolesc..

[23] Malonda E., Llorca A., Mesurado B., Samper P., Mestre M.V. (2019). Parents or peers? Predictors of prosocial behavior and aggression: A longitudinal study. Front. Psychol..

[24] Marcone R., Borrone A., Caputo A. (2021). Peer interaction and social competence in childhood and early adolescence: The affects of parental behaviour. J. Fam. Stud..

[25] Marimon M.P., Alvarez G.Y.O. (2021). Incidence of parental competences in the development of social skills in kids form single children families. Interdisciplinaria.

[26] Tomsik R., Ceresnik M. (2017). Adolescent’s personality through big five model: The relation with parenting styles. AD Alta-J. Interdiscip. Res..

[27] Watson D., Tellegen A. (1985). Toward a consensual structure of mood. Psychol. Bull..

[28] Ciocanel O., Power K., Eriksen A., Gillings K. (2017). Effectiveness of positive youths development interventions: A meta-analysis of randomized controlled trials. J. Youth Adolesc..

[29] Ditcheva M., Vrshek-Schallhorn S., Batista A. (2018). People who need people: Trait loneliness influences positive affect as a function of interpersonal context. Biol. Psychol..

[30] Watson D., Clark L.A., Tellegen A. (1988). Development and validation of brief measures of positive and negative affect: The PANAS scales. J. Personal. Soc. Psychol..

[31] Clark L.A., Watson D. (1991). Tripartite model of anxiety and depression: Psychometric evidence and taxonomicimplications. J. Abnorm. Psychol..

[32] Crawford J.R., Henry J.D. (2004). The Positive and Negative Affect Schedule (PANAS): Construct validity, measurement properties and normative data in a large non-clinical sample. Br. J. Clin. Psychol..

[33] Flores-Kanter P.E., Garrido L.E., Moretti L.S., Medrano L.A. (2021). A modern network approach to revisiting the Positive and Negative Affective Schedule (PANAS) construct validity. J. Clin. Psychol..

[34] Agran M., Hughes C., Thoma C.A., Scott L.A. (2016). Employment social skills: What skills are really valued?. Career Dev. Transit. Except. Individ..

[35] Furlow C.M., Radley K.C., Dart E.H. (2018). What is behavior?. Handbook of Behavioral Interventions in Schools.

[36] Kinnaman J.E.S., Bellack A.S., O’Donohue W., Fisher J.E. (2012). Social Skills. Cognitive Behavior Therapy: Core Principles for Practice.

[37] Caballo V. (2015). Manual de Evaluación y Entrenamiento de las Habilidades Sociales.

[38] Radley K.C., Dart E.H. (2021). What are social skills?. Social Skills Teaching for Individuals with Autism.

[39] Lent R.W., Taveira D.M.C., Figuera P., Dorio I., Faria S., Gonçalves A.M. (2017). Test of the social cognitive model of well-being in Spanish college students. J. Career Assess..

[40] Pannebakker F.D., Van Genugten L., Diekstra R.F., Gravesteijn C., Fekkes M., Kuiper R., Kocken P.L. (2019). A social gradient in the effects of the skills for life program on self-efficacy and mental wellbeing of adolescent students. J. Sch. Health.

[41] Hunter S.B., Barber B.K., Stolz H.E. (2015). Extending knowledge of parents’ role in adolescent development: The mediating effect of self-steem. J. Child Fam. Stud..

[42] Aroca C., Cánovas P., Alba J.L. (2012). Características de las familias que sufren violencia filio-parental: Un estudio de revisión. Educ. Siglo XXI.

[43] Barton A.L., Kirtley M.S. (2012). Gender differences in the relationships among parenting styles and college student mental health. J. Am. Coll. Health.

[44] Lin Y.C., Billingham R.E. (2014). Relationship between parenting styles and gender role identity in college students. Psychol. Rep..

[45] Salavera C., Usán P. (2021). Relationship between social skills and happiness: Differences by gender. Int. J. Environ. Res. Public Health.

[46] Zach S., Yazdi-Ugav O., Zeev A. (2016). Academic achievements, behavioral problems, and loneliness as predictor of social skills among students with an without learning disorders. Sch. Psychol. Int..

[47] Hamama L., Hamama-Raz Y. (2021). Meaning in life, self-control, positive and negative affect: Exploring gender differences among adolescents. Youth Soc..

[48] Seely H.D., Possel P. (2021). Parenting behabior and adolescent affect: Bidirectionality and the role of gender. J. Fam. Issues.

[49] Ato M., Vallejo G. (2015). Diseños de Investigación en Psicología.

[50] Hernández-Guanir P. (1998). Test Autoevaluativo Multifactorial de Adaptación Infantil (TAMAI).

[51] Hernández H., Pelegrín A., Carballo J.L. (2018). Estilos educativos parentales y satisfacción con las expectativas deportivas. EmásF Rev. Digit. Educ. Fis..

[52] Buhrmester D., Furman W., Wittenberg M.T., Reis H.T. (1988). Fivedomains of interpersonal competence in peer relationships. J. Personal. Soc. Psychol..

[53] Salavera C., Usán P. (2018). Adaptación del Cuestionario de Competencia Interpersonal ICQ-15 con población adolescente Hispanohablante. Rev. Iberoam. Diagn. Eval. Psicol..

[54] Cohen J. (1988). Statistical Power Analysis for the Behavioral Sciences.

[55] Aiken L.S., West S.G. (2002). Multiple Regression: Testing and Interpreting Inter-Actions.

[56] Campbell L., Kashy D.A. (2002). Estimating actor, partner, and interaction effects for dyadic data using PROC MIXED and HLM: A user-friendly guide. Pers. Relatsh..

[57] Baron R.M., Kenny D.A. (1986). The moderator-mediator variable distinction in social psychological research: Conceptual, strategic and statistical considerations. J. Personal. Soc. Psychol..

[58] Hayes A.F. (2013). Introduction to Mediation, Moderation and Conditional Process Analysis: A Regression Based Approach.

[59] Tur-Porcar A., Mestre V., Samper P., Malonda E. (2012). Crianza y agresividad de los menores: ¿Es diferente la influencia del padre y de la madre?. Psicothema.

[60] Gámez-Guadix M., Almendros C. (2015). Parental discipline in Spain and in the United States: Differences by country, parent-child gender and education level. Infanc. Aprendiz..

[61] Tur-Porcar A., Mestre V., Llorca A. (2015). Estilos parentales: Análisis psicométrico de dos estudios en población española. Anu. Psicol..

[62] Sorbring E., Rödholm-Funnemark M., Palmérus K. (2003). Boys’ and girls’ perceptions of parental discipline in transgression situations. Infant Child Dev..

[63] Winsler A., Madigan A.L., Aquilino S.A. (2005). Correspondence between maternal and paternal parenting styles in early childhood. Early Child. Res. Q..

[64] Zervides S., Knowles A. (2007). Generational changes in parenting styles and the effect of culture. E-J. Appl. Psychol..

[65] León-del-Barco B., Mendo-Lázaro S., Polo-del-Río M.I., López-Rams V.M. (2019). Parental psychological control and emotional and behavioral disorders among Spanish adolescents. Int. J. Environ. Res. Public Health.

[66] Rosa-Alcázar A.I., Parada-Navas P., Rosa-Alcázar A. (2014). Síntomas psicopatológicos en adolescentes españoles: Relación con los estilos parentales percibidos y la autoestima. An. Psicol..

[67] Aguirre-Dávila E. (2015). Prácticas de crianza, temperamento y comportamiento prosocial de estudiantes de educación básica. Rev. Latinoam. Cienc. Soc. Niñez Juv..

[68] Aziz M., Khan W., Amin F., Khan M.F. (2021). Influence of parenting styles and peer attachment on life satisfaction among adolescents: Mediation role of self-esteem. Fam. J..

[69] Fuentes M.C., García F., Gracia E., Alarcón A. (2015). Los estilos parentales de socialización y el ajuste psicológico. Un estudio con adolescentes españoles. Rev. Psicodidáctica.

[70] Salavera C., Usán P., Antoñanzas J.L., Teruel P., Lucha O. (2017). Affects and personality: A study with university students. Ann. Med. Psychol..

[71] Gómez-Leal R., Megías-Robles A., Gutiérrez-Cobo M.J., Cabello R., Fernández-Berrocal P. (2020). Personal risk and protective factors involved in aggressive behavior. J. Interpers. Violence.

[72] Salavera C., Usán P., Jarie L. (2017). Emotional intelligence and social skills on self-efficacy in Secondary Education students. Are there gender differences?. J. Adolesc..

[73] Trigueros R., Sanchez-Sanchez E., Mercader I., Aguilar-Parra J.M., López-Liria R., Morales-Gázquez M.J., Fernández-Campoy J.M., Rocamora P. (2020). Relationship between Emotional Intelligence, Social Skills and Peer Harassment. A Study with High School Students. Int. J. Environ. Res. Public Health.

[74] Kong F., Gong X., Sajjad S., Yang K., Zhao J. (2019). How is emotional intelligence linked to life satisfaction? The mediating role of social support, positive affect and negative affect. J. Happiness Stud..

[75] Pinquart M., Gerke D.C. (2019). Associations of parenting styles with self-esteem in children and adolescents: A Meta-analysis. J. Child Fam. Stud..

[76] Yun B.X., Thing T.S., Hsoon N.C. (2019). A quantitative study of relationship between parenting style and adolescent’s self-esteem. Adv. Soc. Sci. Educ. Humanit. Res..

[77] García F., Gracia E. (2009). Is always authoritative the optimum parenting styles? Evidence from Spanish families. Adolescence.

[78] Martínez I., García J.F. (2007). Impact of parenting styles on adolescents self-esteem and internalization of values in Spain. Span. J. Psychol..

[79] Bush K.R., Peterson G.W., Peterson G.W., Bush K.R. (2013). Parent-child relationships in diverse contexts. Handbook of Marriage and the Family.

[80] Möller E.L., Nikolić M., Majdandžić M., Bögels S.M. (2016). Associations between maternal and paternal parenting behaviors, anxiety and its precursors in early childhood: A meta-analysis. Clin. Psychol. Rev..

[81] García O.F., Serra E., Zacarés J.J., García F. (2018). Parenting styles and short- and long-term socialization outcomes: A study among Spanish adolescents and older adults. Psychosoc. Interv..

[82] Hadfield K., Amos M., Ungar M., Gosselin J., Ganong L. (2018). Do changes to family structure affect child and family outcomes? A systematic review of the Instability Hypothesis. J. Fam. Theory Rev..

[83] Latsch D.C., Nett J.C., Humbelin O. (2017). Poly-victimization and its relationship with emotional and social adjustmente in adolescence: Evidence from a National Survey of Switzerland. Psychol. Violence.

[84] Lawall A.R., Tram J.M., Kumar N. (2021). The impact of parenting styles on subsequent parenting styles in sons. Fam. J..

[85] Chan T.W., Koo A. (2011). Parenting style and youth outcomes in the UK. Eur. Sociol. Rev..

[86] Milevsky A., Schlechter M., Netter S., Keehn D. (2007). Maternal and paternal parenting styles in adolescents: Associations with self-esteem, depression and life-satisfaction. J. Child Fam. Stud..

[87] Liu X. (2020). Parenting styles and health risk behavior of left-behind children: The mediating effect of cognitive emotion regulation. J. Child Fam. Stud..

[88] Pacheco M., Osorno G.Y. (2021). Incidencia de competencias parentales en el desarrollo de habilidades sociales en hijos únicos. Interdisciplinaria.

[89] Ayarza Y., Villalobos S., Bolívar L., Ramos N., Rentería K., Arias A., Vanegas M. (2014). Las familias en Urabá: Estado del arte sobre familias, tipologías, crianza y sus transformaciones. Educ. Humanismo.

